# Combining dispersal, landscape connectivity and habitat suitability to assess climate-induced changes in the distribution of Cunningham’s skink, *Egernia cunninghami*

**DOI:** 10.1371/journal.pone.0184193

**Published:** 2017-09-05

**Authors:** Benjamin Y. Ofori, Adam J. Stow, John B. Baumgartner, Linda J. Beaumont

**Affiliations:** 1 Department of Biological Sciences, Macquarie University, North Ryde, Macquarie Park, NSW, Australia; 2 Department Animal Biology and Conservation Science, University of Ghana, Legon-Accra, Ghana; University of New England, AUSTRALIA

## Abstract

The ability of species to track their climate niche is dependent on their dispersal potential and the connectivity of the landscape matrix linking current and future suitable habitat. However, studies modeling climate-driven range shifts rarely address the movement of species across landscapes realistically, often assuming “unlimited” or “no” dispersal. Here, we incorporate dispersal rate and landscape connectivity with a species distribution model (Maxent) to assess the extent to which the Cunningham’s skink (*Egernia cunninghami*) may be capable of tracking spatial shifts in suitable habitat as climate changes. Our model was projected onto four contrasting, but equally plausible, scenarios describing futures that are (relative to now) hot/wet, warm/dry, hot/with similar precipitation and warm/wet, at six time horizons with decadal intervals (2020–2070) and at two spatial resolutions: 1 km and 250 m. The size of suitable habitat was projected to decline 23–63% at 1 km and 26–64% at 250 m, by 2070. Combining Maxent output with the dispersal rate of the species and connectivity of the intervening landscape matrix showed that most current populations in regions projected to become unsuitable in the medium to long term, will be unable to shift the distance necessary to reach suitable habitat. In particular, numerous populations currently inhabiting the trailing edge of the species’ range are highly unlikely to be able to disperse fast enough to track climate change. Unless these populations are capable of adaptation they are likely to be extirpated. We note, however, that the core of the species distribution remains suitable across the broad spectrum of climate scenarios considered. Our findings highlight challenges faced by philopatric species and the importance of adaptation for the persistence of peripheral populations under climate change.

## Introduction

Climate change presents a major threat to global biodiversity and ecosystem services, upon which humans depend [1, 2]. Widespread evidence suggests that climate change has already and will continue to cause changes in species’ distributions [3–5], alterations in phenology [6–11], physiology [1], morphology [12, 13], demography and community composition [14–16], and the nature of ecological interactions [17, 18]. Species level extinctions have been reported [19, 20], with rates of extinction projected to accelerate as climate change intensifies [20–24]. To minimize the loss of global biodiversity, it is important that conservation practitioners, resource managers and decision-makers adapt their management practices and environmental policies towards amelioration of the impacts of climate change [25–27]. To do this effectively, a greater capacity to model the impacts of climate change on species distributions is needed [28, 29].

Species distribution models (SDMs) (also called habitat suitability models, bioclimatic models or climate niche models) are frequently used to predict the potential distribution or redistribution of species under climate change. SDMs have their foundation in ecological niche theory and are based on the principle that a species’ distribution correlates with landscape variables that are related to the physiological tolerance or resource requirements [30–32]. Depending on whether the fundamental niche or realized niche is characterized, SDMs can be broadly grouped into two: mechanistic or process-based SDMs and correlative SDMs [33–36].

Mechanistic SDMs are based on species’ functional traits and physiological constraints [34–36]. They assume that all species have physiologically-based environmental constraints to their abundance and distribution. These physiological processes are believed to be strongly tied to the flow of mass and energy as individuals interact with the environment [29, 37]. Mechanistic SDMs rely on a set of morphological, behavioural, physiological and other life-history traits and abiotic variables, such as temperature, solar radiation, wind speed and precipitation to infer species distribution. For this reason they are believed to model species’ fundamental niche and provide more accurate projections of species distributions because they are transferable to novel environment and climate [34, 35]. However, the use of mechanistic models is often prohibited by the requirement for a detailed understanding of species’ physiology, behaviour and the complex processes and interactions that determine an organism’s performance [38].

Correlative SDMs model a species’ distribution by relating its occurrence data to spatial environmental data [32, 36]. These tools estimate the statistical relationship between environmental characteristics and species’ occurrence patterns, and identify suitable habitats under past, present or future environmental conditions [33, 39]. Unlike mechanistic approaches, correlative SDMs rely heavily on the assumption of the modeled species equilibrium with the environment and occurrence sample’s adequacy in reflecting suitable conditions. This potential drawback is balanced by correlative methods’ capacity to implicitly capture latent environmental variables that affect habitat suitability (e.g., species interactions) via relationships between these variables and the included environmental predictors. Correlative SDMs have become mainstream tools for projecting climate-driven range changes because they require less data, which are readily available or easily obtained in the field and are easy to implement within freeware packages [33]. Correlative SDMs have been used to assess potential climate-driven range shifts and extinction risks for many terrestrial and aquatic invertebrates, vertebrates and plants [23, 40–47].

The capacity of organisms to move across landscapes is critical in determining their ability to locate suitable habitats [48, 49]. Yet, many correlative SDM studies do not account for dispersal, and those that do, frequently assume that species have either “unlimited” or “no” dispersal [23, 38, 50]. Some studies have, however, used alternate approaches to incorporate more realistic estimates of dispersal [51]. The most straight-forward of these approaches adopts a colonization potential based on the “nearest-neighbour” concept, where grid cells that become climatically suitable can be colonized only if a neighboring cell is already occupied [52–54]. Others have applied a Euclidean distance equal to the average dispersal distance of the study species, to create buffers around the current distribution. Projected suitable habitat outside the buffer zone is presumed to be unreachable by the species [54, 55]. In contrast, relatively more-complex models fit statistical functions, such as dispersal kernels [56], or involved spatially explicit metapopulation models [57–59].

Although these approaches have made substantial contributions towards incorporating realistic estimates of dispersal into the output of SDMs, they do not address the effects of landscape structure and features on the behaviour and movement rates of organisms. Landscapes are spatially heterogeneous, and their degradation and fragmentation, due to human activities [60], can dramatically impede the spread of organisms [61–64]. Consequently, the output of SDMs may be misleading if landscape connectivity and its effects on dispersal are ignored. This can have considerable implications for spatial conservation planning and prioritization.

To address this discrepancy, we combine dispersal rates and a landscape connectivity model that accounts for fine-scale habitat heterogeneity and barriers to movement, with the output of a correlative SDM to assess climate-induced range shifts for Cunningham’s skink (*Egernia cunninghami*). We used a genetic isolation-by-distance model to obtain an estimate of dispersal and a least-cost path (LCP) analysis to model the functional connectivity of the intervening landscape matrix. LCPs use algorithms within a geographical information system (GIS) to identify landscape features and cover types that a moving organism prefers or avoids, to locate the route that provides the lowest cumulative resistance between source and destination [65]. LCPs have been used extensively for spatial conservation planning because they require less data than other connectivity models and are readily calculated [66]. Specifically, we asked the following questions: (i) To what extent will the distribution of suitable habitat be altered under different future climate trajectories? (ii) To what extent will suitable habitat be located within protected areas in the future? (iii) What are the effects of spatial resolution on the size and configuration of future suitable habitat? (iv) What proportion of inhabited grid cell that area projected to become unsuitable in the future is within reachable distance to the nearest suitable grid cell?

## Methodology

### Species occurrence data

Cunningham’s skink (*Egernia cunninghami*) is a common and widespread scincid lizard endemic to southeastern Australia. Its distribution extends from south-east Queensland through New South Wales to the Great Dividing Range in northeastern Victoria, with disjunct populations in the Mount Lofty Ranges in South Australia. The species typically prefers to live communally in relatively large and deep crevices within rock outcrops, in hollow logs or under large slabs of rock in forest, open woodland or cleared areas [67–69]. Occurrence records were obtained from the Global Biodiversity Information Facility (www.gbif.org, accessed March 2014) and Atlas of Living Australia (www.ala.org.au, accessed March 2014). A total of 852 unique occurrence points were available for modeling after removing duplicate and spatially unreliable records.

### Bioclimatic data

Current and future climate data were derived from the NSW and ACT Regional Climate Modelling (NARCliM) project [70]. These data are described in full by Hutchinson and Xu [71] and are outlined briefly below. Gridded data describing current climatic conditions (i.e., 1990–2009) were derived from Bureau of Meteorology observations, and served as a basis upon which future change grids were applied. Generation of these surfaces involved calculation of elevation dependent climate surfaces [72] and transformation to standard bioclimatic variables [73] at 0.01° and 0.0025° spatial resolutions. Future projections were generated by four global climate models (GCMs): MIROC3.2-medres [74], ECHAM5/MPI-OM [75], CGCM3.1-T47 [76] and CSIRO-Mk3.0 [77]. In general, CGCM3.1 is a hot/wet scenario, MIROC3.2 is a warm/wet scenario, ECHAM5 is a hot/similar precipitation scenario, and CSIRO-Mk3.0 is a warm/dry scenario, relative to the period 1990–2009 [78]. These were dynamically downscaled to 0.1° resolution by Evans and Ji [78] for south-eastern Australia using the Weather and Research Forecasting (WRF) Regional Climate Model, for both 2020–2039 (i.e., “2030”) and 2060–2079 (i.e., “2070”). Three alternate parameterizations of the WRF model (hereafter R1, R2, and R3), were used for the downscaling, resulting in 12 climate scenarios for each future time period. The NARCliM project assumed the A2 emissions scenario [79], which approximates the relative forcing and mean temperature trajectories of the RCP8.5 scenario [80].

We projected the above current and future climate data to the Australian Albers Equal Area coordinate system at resolutions of 1 km (for 0.01° data) and 250 m (for 0.0025° data), with standard parallels chosen to minimize distortion across the study area [81]. Since NARCliM was restricted to the time periods centered on 2000, 2030 and 2070, we calculated data for the intervening decades (i.e. 2010, 2020, 2040, 2050, 2060) via linear interpolation using R v.3.1.2 [82].

We used a suite of five predictor variables that have been shown to model the distribution of reptiles adequately [83]. These included (i) annual mean temperature, (ii) temperature seasonality, (iii) maximum temperature of the warmest month, (iv) minimum temperature of the coldest month and (v) annual precipitation. Cunningham’s skink dwells in crevices of granite rock outcrops, which provide thermal buffering at fine spatial scales [84, 85]. Hence, we included an index of rock cover (Weathering Intensity Index) [86] as a static predictor variable. We consider this an important addition because the presence of rock-outcrops with suitable crevices will largely determine range filling under climate change.

### Species distribution modeling

The distributions of current and future habitat were projected using Maxent run in R v.3.12 [82]. Maxent is a machine learning algorithm that estimates species-environment relationships from spatial environmental data and species’ occurrence records [87, 88]. We used Maxent because of its high predictive performance, computational efficiency and ease of use [87, 88]. Indeed, many recent studies comparing the performance of different SDM algorithms across various taxa and geographic locations including Australia have shown that Maxent is one of the best performing correlative SDMs [89–93].

We fitted the model using different combinations of the linear, quadratic, product, threshold and hinge features, varying regularization to control how tightly the model fitted the given occurrence points. Response curves were visually inspected to ensure that the estimated relationships were ecologically realistic. The most realistic model settings (determined by the smoothness of the response curve) for the Cunningham’s skink were the linear, product and quadratic features with a regularization multiplier of 1.5. Model performance has been shown to be low when background points are taken from either a too narrow or too broad region [94]. Therefore, to reduce under- or over-prediction, background records were randomly sampled from grid cells within 100 kilometers of occurrence localities.

Model performance was evaluated using the area under the Receiver Operating Characteristic curve (AUC) and the True Skill Statistic (TSS) using 10-fold cross-validation with randomly selected folds. AUC scores range from 0 to 1. Values of 1 indicate perfect model accuracy while 0.5 suggests that model performance is no better than random. TSS, also known as the Hanssen-Kuipers discriminant, takes into account both omission and commission errors, and success as a result of random guessing [95]. For a confusion matrix, TSS is defined as the sum of sensitivity and specificity minus one (sensitivity + specificity– 1). TSS scores range from -1 to 1, where 1 indicates perfect agreement between test data and model predictions, and scores of 0 or less indicate performance no better than random [95]. Variable predictive ability and importance to the model were assessed by percentage contribution and jack-knifing. At both spatial resolutions (1 km and 250 m), the final model was fitted using all occurrence data, and habitat suitability maps were generated by projecting these models onto predictor data for the current and six future time slices (2020, 2030, 2040, 2050, 2060 and 2070). Future projections were constrained using a buffer of 300 km around the species’ current distribution to ensure that the model did not predict climatically suitable habitats in areas that the Cunningham’s skink could not reach unaided.

The projected continuous habitat suitability maps were transformed into binary suitable and unsuitable habitat using the maximum training sensitivity and specificity logistic threshold [96]. This threshold was used because it maximizes the combined rate of correctly predicted presences (sensitivity) and correctly predicted absences (specificity, but see [97, 98]) and has been shown to perform very well [99], including when using presence-background data [98]. We calculated the change in climatically suitable habitat between the current and future climate scenarios as the percentage change in the number of suitable grid cells. We also calculated the percentage overlap between the current and future suitable habitat and the percentage of suitable habitat lost or gained within protected areas. For the latter, we obtained a GIS layer of the Australian Protected Area network from the Collaborative Australian Protected Area Database (CAPAD 2014; available at http://www.environment.gov.au/land/nrs/science/capad).

### Estimating annual dispersal distance

Previous capture-mark-recapture (CMR) studies of the Cunningham’s skink recorded 70.1 m as the longest dispersal distance over four years [100]. Although dispersal in skinks is generally limited, this measure may be an underestimate of the species’ dispersal capacity because of its life-history (i.e. high retreat site fidelity) and the limitation of CMR in capturing long-distance dispersal events relevant for colonization of new habitats [101]. For this reason, we estimated annual dispersal distance using the genetic isolation-by-distance (IBD) model which relates matrices of genetic distance between individuals to matrices of geographical distance [102, 103]. According to Wright [103], the “neighbourhood” is defined by the average distance between the natal and breeding sites of the study species. The neighbourhood encompasses the spatial extent within which gene flow is random. In two-dimensional space, the neighbourhood size (NS) is equal to 4πρσ^2^, which is equal to the inverse of the regression slope between a multilocus estimator of individual pairwise genetic distances [F_ST_/(1-F_ST_)] and geographic distance, where ρ is the population density, σ^2^ is the variance of dispersal per generation and F_ST_ is the pairwise genetic distance between individuals [104].

This estimate of dispersal is potentially biased by the scale of analysis, as genetic structure may change with scale. We attempted to measure average annual dispersal distance for *E*. *cunninghami* using genetic data from four locations, Armidale, Bathurst, Crookwell and Sydney, in order to assess the uncertainty associated with the dispersal estimate. However, estimation of dispersal using genetic data from Bathurst, Crookwell and Sydney was unsuccessful because the samples were too small and geographically clumped. Therefore, we estimated average annual dispersal distance using genetic data from the Armidale population only [105]. We used a generation time of five years and population density of 120 individuals per km^2^_,_ as recorded by previous studies [100, 106].

### Modeling functional connectivity

We created a resistance map of the study area using land-use/cover raster map of Australia [107] at 1 × 1 km resolution. Resistance values of each cover type were based on expert knowledge. We applied the Delphi method [108] to calibrate the cost-surface representation of the landscape. We identified a group of seven experts with substantial knowledge of the ecology of Australian reptiles, and asked them to rank the different land cover types in our study area based on their potential cost to the movement of Cunningham’s skinks. Cover types likely to be avoided by the species because they impede movement, are a total barrier to movement, or expose the species to danger, were given higher cost values than those that facilitate movement. Cover types considered as total barriers were awarded a resistance score of 100, while those through which the species readily moves were awarded a score of 1. Originally, the collated results were to be returned anonymously to each expert, following which ranking would take place a second time. However, because the scores from the experts were very similar, we created the resistance surface using the mean scores from the first iteration (S1 Table).

The resistance map was created using the Resistance and Habitat Calculator in the Gnarly Landscape Utilities ArcGIS toolbox [109]. Least-cost paths from occupied cells that were projected to become unsuitable to the nearest cells that became or remained suitable in the subsequent time horizon were modeled using the landscape connectivity model in the SDMtoolbox package [110], implemented in ArcGIS v10.2.1 (ESRI Inc., Redlands CA, USA, 2013). Given computational processes and software challenges of modeling connectivity at fine resolution over large spatial scales, we modeled landscape connectivity at 1 × 1 km only (but see [111], for an example of high of high resolution connectivity modeling at a sub-continental scale).

Ideally, we would compute LCPs for all occupied grid cells projected to become unsuitable under any of the 12 climate scenarios, for each decade. However, because of computational limitations, we restricted the analysis to only occupied grid cells that were projected to become unsuitable under *all* climate scenarios for each decadal time interval. We suggest this is a reasonable approach, as all future scenarios are assumed equally plausible [78, 112] and populations projected to have no suitable habitat under all 12 scenarios will be most vulnerable to climate change.

### Evaluation of the capacity of Cunningham’s skink to track its climate niche

Extant populations occupying grid cells projected to become unsuitable in future were considered capable of tracking their climate niche if the least-cost path distance to the nearest suitable location was less or equal to the mean decadal dispersal distance of the species.

## Results

### Distribution of current suitable habitat

**Fig 1:**
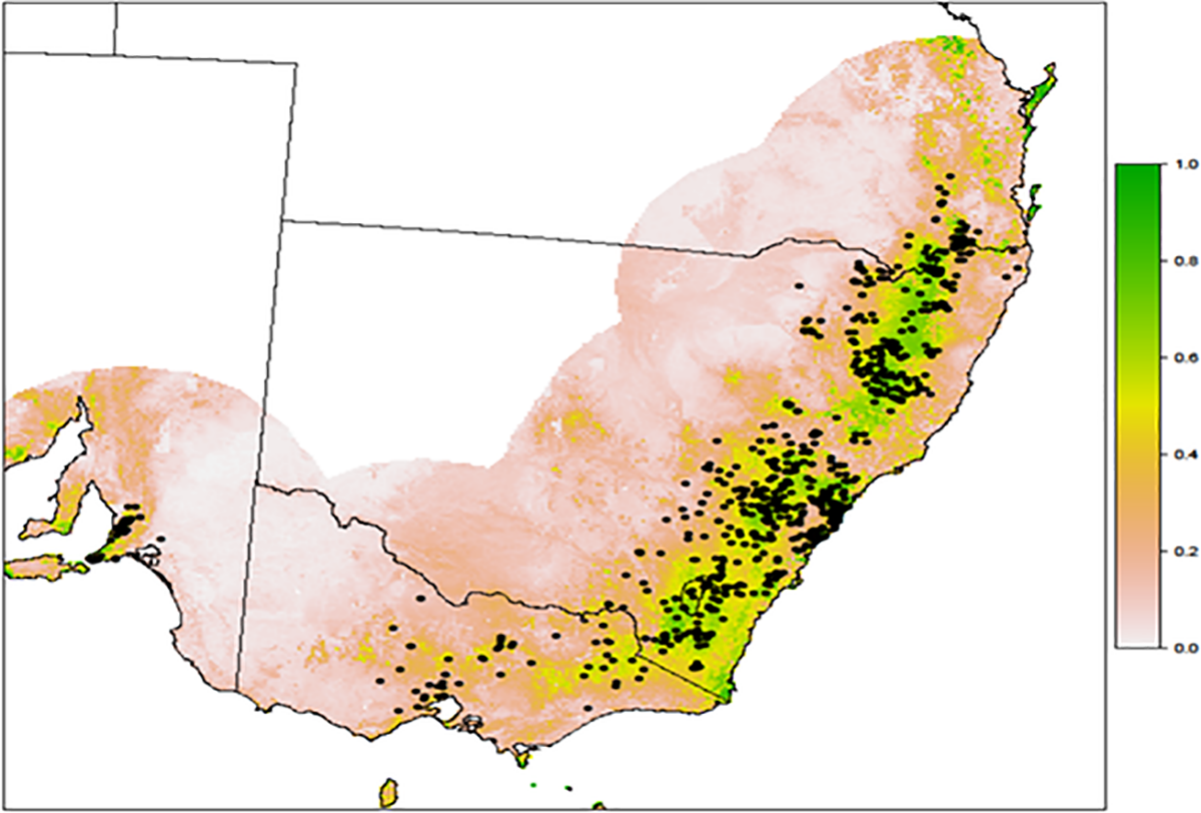
**Projected current habitat suitability for Cunningham’s skink modelled using Maxent (A = logistic map, B = thresholded map using maximum training sensitivity and specificity logistic threshold of 0.42.** For the logistic map, suitability ranges from 0 (least suitable) to 1 (most suitable). Black points indicate species occurrence records.

Models had high predictive capacity at both spatial resolutions, with an average AUC of 0.818 and 0.793 and TSS of 0.492 and 0.451 for the 1 × 1 km and 250 × 250 m, respectively. The amount of currently suitable habitat projected at 1 × 1 km (15,951,800 ha) was higher than that projected at 250 × 250 m (14,267,150 ha). Generally, the projected current distribution of suitable habitat was consistent with the species’ known range. However, the range margin of the projected distribution was situated slightly to the north of the northernmost populations in southern Queensland. Also, areas in southeastern New South Wales (NSW) and some regions in Southern Australia where the species has not been recorded were projected to be highly suitable (Fig 1). After transforming the projected current suitability map into binary suitable and unsuitable habitat using the maximum training sensitivity and specificity logistic threshold (0.42), 74% (632 of 852) of the occurrence points were within suitable habitat at 1 × 1 km and 72% (613 of 852) at 250 × 250 m. Maximum temperature of the warmest month contributed most (45% for the 1 × 1 km and 47% for the 250 × 250 m resolutions) to the models of habitat suitability.

### Projected changes in climatically suitable habitat

**Fig 2:**
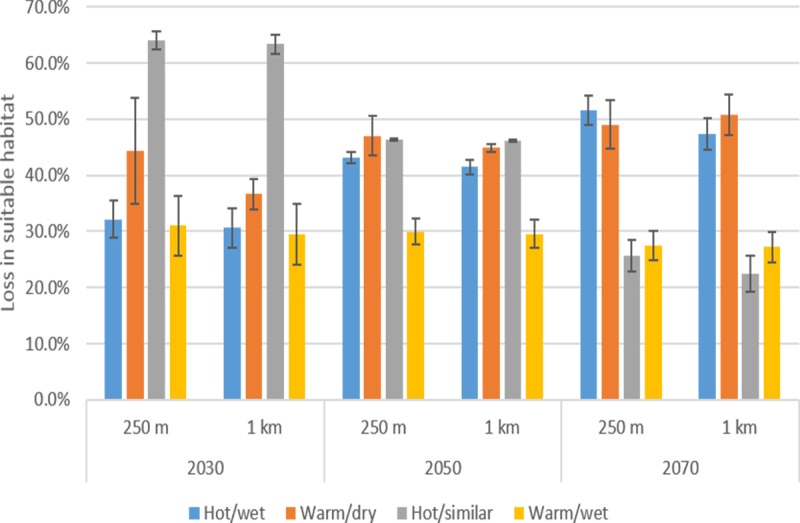
Percentage loss in climatically suitable habitat of Cunningham’s skink projected at 1 km and 250 m under four contrasting but equally plausible future climate scenarios (hot/wet, warm/dry, hot/similar precipitation and warm/wet) at 2030, 2050 and 2070. Each climate scenario was parameterized using three alternatives of the Weather and Research (WRF) Regional Climate model (R1, R2 and R3), hence values of vertical lines represent standard errors of values across all grid cells.

The area of climatically suitable habitat for Cunningham’s skink was projected to decline by 22.5–63% for the 1 × 1 km and 26–64.0% for 250 × 250 m resolutions by 2070, depending on the climate trajectory and time horizon (Fig 2). In general, suitable habitat was progressively lost over time (from current to 2070), at both spatial resolutions and under most climate scenarios. The exception was the hot/similar precipitation scenario, where the proportion of suitable habitat projected to be lost was highest in 2030 then declined (Fig 2). The projected percentage loss of suitable habitat at 1 × 1 km and 250 × 250 m for the respective time periods did not differ significantly across climate scenarios (Fishers Exact Test: df = 198; *p* > 0.1).

Under all climate scenarios, habitat was progressively lost from the northern range margins in southeastern Queensland and northeastern NSW, and the western margins toward southeastern NSW, although the core of the skinks’ range was projected to remain stable. Under all scenarios, new habitat was gained around the southern region of South Australia and some regions in southeastern NSW, particularly from 2050 to 2070 (S1 Fig).

### Changes in suitable habitat within protected areas

**Fig 3:**
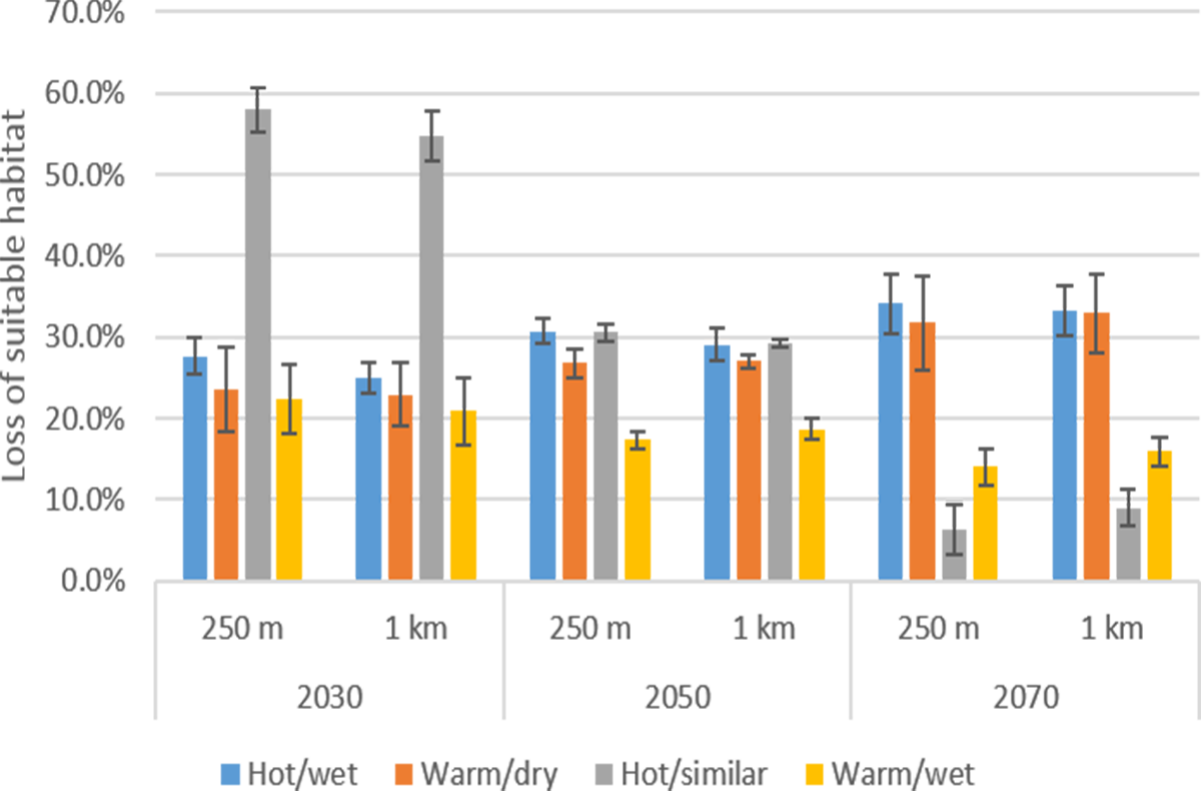
Percentage loss in climatically suitable habitat of Cunningham’s skink within protected areas projected at 1 × 1 km and 250 × 250 m under four contrasting but equally plausible future climate scenarios (hot/wet, warm/dry, hot/similar precipitation and warm/wet) at 2030, 2050 and 2070. Each climate scenario was parameterized using three alternatives of the Weather and Research (WRF) Regional Climate model (R1, R2 and R3), hence values of vertical lines represent standard errors of values across all grid cells.

The area of current suitable habitat projected to be within protected areas (PAs) at 1 × 1 km and 250 × 250 m resolutions was 4,696,500 and 4,226,777 hectares, respectively. At both spatial resolutions, the projected loss of suitable habitat (relative to 2000) increased slightly over time under the hot/wet and warm/dry scenarios, but declined from 2030 onwards under the hot/similar precipitation and warm/wet scenario (Fig 3). At 250 × 250 m resolution, the loss in suitable habitat ranged from 27.7% (SD = 3%) by 2030, to 34.1% (SD = 6%) by 2070 under the hot/wet condition, and from 23.5% (SD = 5%) to 31.7% (SD = 10%) under the warm/dry scenario. Under the hot/similar precipitation scenario, the highest projected loss of suitable habitat within PAs at both spatial resolutions (58% at 250 × 250 m and 55% at 1 × 1 km) occurred by 2030, and the lowest (6% at 250 × 250 m and 9% at 1 × 1 km) by 2070. Similarly, under the warm/wet condition, the area projected to become unsuitable was highest (22% at 250 × 250 m and 21% at 1 × 1 km) by 2030, and lowest (14% at 250 × 250 m and 16% at 1 × 1 km) by 2070. Again, the percentage loss of suitable habitat within PAs projected at 1 × 1 km and 250 × 250 m for the respective time periods under all the climate trajectories did not differ significantly (Fishers Exact Test: df = 198; *p* > 0.1).

### Dispersal rate and ability of the species to track its climate niche

Using a population density of 120 individuals per km^2^_,_ the isolation-by-distance model estimated the average dispersal rate of Cunningham’s skink to be 4.3 km per generation (i.e. 5 years). Hence, occupied grid cells projected to become unsuitable in the next decade must be within a distance of 8.6 km in order for individuals or populations inhabiting them to be able to track spatial shifts in their climate niche.

By 2070, 63 (10%) of the 632 occupied grid cells were projected to become unsuitable under *all* climate trajectories considered. Eight occupied grid cells were consistently projected to be unsuitable by 2020. As the cost-adjusted distance between these cells and the nearest suitable cells by 2030 exceed the skink’s average decadal dispersal distance by 1–35.5 km (S2 Table), it is unlikely that any of the individuals/populations inhabiting these grid cells will be able to track their climate niche. By 2030, an additional 16 grid cells currently containing populations of Cunningham’s skink were projected to become unsuitable, and only five of these will have suitable grid cells within the average decadal dispersal distance of the skink. By 2040, 2050 and 2060, an additional 23, 12 and eight occupied grid cells, respectively, were projected to be unsuitable. For all of these, cells with suitable habitat are at a distance greatly exceeding the skink’s capacity for dispersal (17.6–92.3 km) (S2 Table). By 2070, no additional occupied grid cell was projected to lose its suitability under all the climate trajectories used. Thus, of the 63 occupied grid cells projected to be unsuitable under all climate scenarios for 2070, individuals or populations inhabiting 58 (92%) will be unable to track their climate niche.

## Discussion

Our projections show that climate change could cause substantial declines in the spatial extent of suitable habitat for Cunningham’s skink, with range margins progressively retracting towards the core of its current distribution along the Great Dividing Range (GDR) in New South Wales (NSW). Critically, numerous populations currently inhabiting the trailing edge of the species’ range are highly unlikely to be able to disperse fast enough to track range shifts. Suitable habitats within protected areas, particularly those located along the species’ range periphery, are projected to decline over time. However, core areas of the species’ distribution along the GDR retained their suitability across a broad spectrum of plausible climate scenarios (spanning futures described as warm/wet, warm/dry, hot/wet, hot/similar precipitation, relative to 2000).

Whilst the size of suitable habitat is projected to decline under all future climate scenarios considered, the most severe decline (of 63%) was projected to occur under the hot/similar precipitation scenario by 2030. Under this scenario, the lowest decline was projected for the decade 2060–2070 (23%), primarily due to gains in suitable habitat after 2030 in areas around Mount Lofty in Southern Australia and regions in north-western NSW.

We estimate that 63 of the 632 grid cells with extant populations will have no suitable habitat under any of the 12 climate scenarios. Of these, only five occupied grid cells were situated near suitable habitat within the dispersal distance of the species. Our data suggest that for populations inhabiting 92% of grid cells projected to become unsuitable, the skink’s average dispersal rate (8.6 km per decade) is insufficient for the climate niche to be tracked. This is particularly true for populations inhabiting grid cells located at the range margins, suggesting that climate-induced decadal shifts in the species’ range will outpace its dispersal capacity. This highlights the challenge faced by the Cunningham’s skink, and other philopatric species under climate change.

The modeled current distribution of suitable habitat corresponded with the actual distribution of the species. Projected current habitat was, however, slightly farther north than the known range margin, and some areas within southeastern NSW where the species has not been recorded were also projected to be suitable. This indicates that factors other than climate, such as dispersal and biotic interactions (e.g. competition, predation and parasitism) may be preventing the species from attaining its full potential distribution [36, 113]. The incomplete range filling could also mean that the species may not be in equilibrium with the current climate throughout its range [30, 114]. Our results are consistent with previous studies assessing the potential impacts of climate change on Australian reptiles. For example, future declines in suitable habitat have been projected for the endangered Australian broad-headed snake, *Hoplocephalus bungaroides* [42]. Under a hot/dry scenario, the species is projected to lose over 80% of its suitable habitat by 2070 [42]. Fifty-three Australian elapid snakes and 275 skinks have also been projected to experience range contractions by 2050, with some of these species projected to lose their entire range under hot/dry conditions [83, 115]. Similar results have been reported for European species. For example, in the Iberian Peninsula, the ranges of many reptiles have been projected to contract by 2050 and 2080, with more severe declines projected under hot/dry conditions [30, 116].

The spatial resolution at which species’ distributions are modeled can affect estimated declines or gains in climatically suitable habitat [39, 50, 62]. Coarse resolution grid cells may contain a wide range of environments and microclimates, some of which might be suitable for species at their thermal margins [117, 118]. Thus, models fitted with predictors scaled at a coarse resolution may not reflect locally suitable microclimates within which species persist at range margins [118, 119]. Bias in model fitting could lead to over- or under-estimation of species’ suitable habitat. Fitting SDMs with fine-resolution predictor variables may be more accurate than modeling at coarse-resolution depending on the species [120]. For instance, Gillingham, Huntley [118] noted that models fitted with coarse-resolution predictors were associated with higher prediction errors. They also found that the spatial extent of areas projected to remain or become suitable in future varied across different resolutions. Similarly, higher rates of retraction at range margins were found when habitat suitability was modeled at 1 km^2^ compared to 100 km^2^ [44]. In the present study, greater declines in the area of future suitable habitat were recorded when modeled at a resolution of 1 × 1 km than when modeled at 250 × 250 m. It is worth noting however, that the proportional change (i.e., the ratio of the amount of future suitable habitat to the amount of current suitable habitat) and the spatial distribution of future suitable habitats were comparable across resolutions.

Several studies have demonstrated considerable variability in the performance of different correlative SDM algorithms that can compromise conservation decisions. For example, in their analysis of SDM performance among prediction maps for 15 rare vertebrate species in the southeastern USA using seven potential sources of uncertainty: model algorithms, climate data sets, model domain, species presences, variable collinearity, CO2 emissions scenarios and general circulation models, Watling, Brandt [92] noted that the choice of modeling algorithm presented the greatest source of uncertainty in SDM performance. Pearson, Thuiller [121] examined agreement about range prediction by nine SDM algorithms for four South African plant species using a standardized data set under current and future climates. They found that predicted distribution changes varied from about 90% loss to over 300% gain for one species, with equally wide variability in distribution change was predicted for the other species. Beaumont, Graham [89] assessed the tendency for alternative model algorithms to predict extreme range changes and found that some models were more likely to predict extreme distribution changes than others. Distance-based models were less likely to predict considerable increases in size of suitable habitat. Random Forest and Surface Range Envelopes in particular, were more likely to predict complete loss of current habitat, while Generalized Additive and Linear models rarely predicted range extinction. Classification Tree Analysis but not Maxent, predicted future habitat completely disjunct from current habitat than expected more often [89].

Ensemble forecasting of species distributions has been proposed as a potential solution to the discrepancies in the prediction of alternative SDM algorithms [122]. This approach involves the use of multiple models within a single framework. The tenet of ensemble forecast is that combined forecasts yield a lower mean error than any of the constituent individual forecasts [122]. Although Maxent has been shown to perform very well under Australian climate conditions in comparison to other models [89, 91], we may have obtained different distribution changes if we used different model algorithm or an ensemble of alternative model algorithms. Thus, our results should be interpreted cautiously.

Assigning appropriate resistance values to the different cover types and landscape features is crucial in connectivity modeling. We employed expert opinion to estimate resistance values because of a lack of empirical data on the species movements, such as travel path, relocation or adequate genetic data from most of the species range. Our approach is therefore subjective and might not necessarily reflect how the species views the landscape [66]. Nonetheless, expert opinion provides a good approximation of the resistance values of environmental variables and is the only option until empirical data becomes available [66].

Our results show that most populations occupying the range margins and other locations projected to become unsuitable in the future may not be able to move to suitable locations. If our findings hold true for lizards and reptiles in general, then this has dire consequences for conservation. Equally, being able to move to climatically suitable habitat might not necessarily guarantee a population’s long-term survival and avoidance of local extinction under climate change. Ofori, Beaumont [105] have shown that Cunningham’s skinks show strong genetic structuring and signatures of selection across its range. Convergence of populations that are adapted to different local conditions at the same grid cells and exchange of genetic materials between them may be problematic as this can disrupt locally adapted gene complexes and result in outbreeding depression [123]. Also, the fitness of populations may decline if the conditions they experience at the new locations are different from those to which they are adapted. For example, populations may encounter new predators, competitors and/or disease, which could dramatically reduce their size and reproductive output.

Further research using other species is needed to ascertain the transferability of our method and the generality of our results. Furthermore, there is a need to determine whether lizards have sufficient variation in adaptive traits to enable those that are unable to track their climate niche to evolutionarily adapt to climate change in situ.

## Supporting information

S1 Fig Changes in climatically suitable habitat of the Cunningham’s skink over time projected (using Maxent) at 1 km × 1 km resolution under the different climate trajectories. The warm colour shows areas projected to be highly suitable.
(PDF)

S1 Table Mean resistance scores for the land-use and land cover types in the study area as perceived by seven herpetologists (experts). Scores are based-on the tendency of and ease with which Cunningham’s skinks will move through the different cover types.
(PDF)

S2 Table Least-cost distance (in meters) between occupied suitable grid cells that were projected to become climatically unsuitable and the nearest grid cell projected to retain its suitability or to become suitable for each decadal time interval. (PDF)
