# Supplementary figures and images for: Combining dispersal, landscape connectivity and habitat suitability to assess climate-induced changes in the distribution of Cunningham’s skink, Egernia cunninghami
Source: PLoS One. 2017 Sep 5;12(9):e0184193. doi: 10.1371/journal.pone.0184193 (PMC5584964; doi:10.1371/journal.pone.0184193)

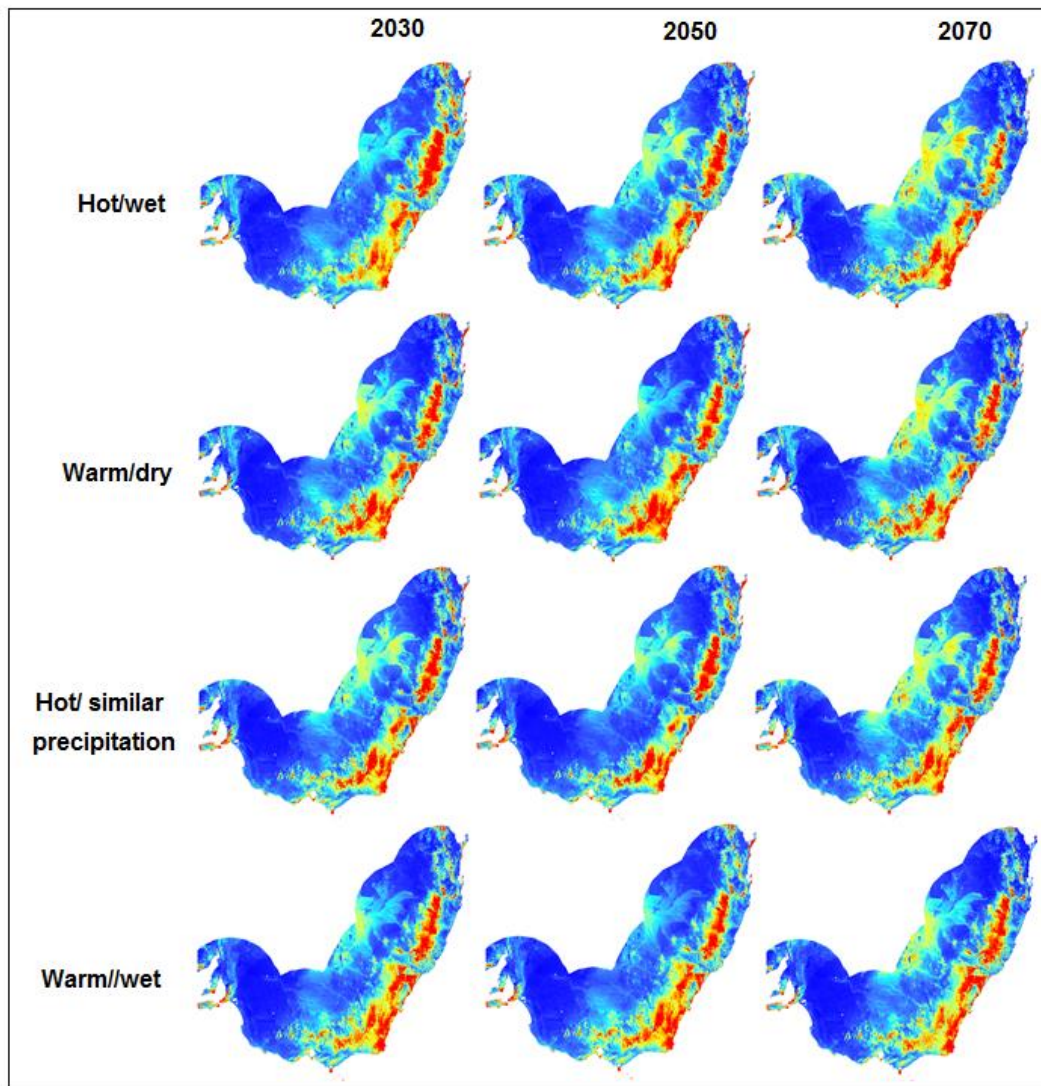

Supplement: S1 Fig — The warm colour shows areas projected to be highly suitable. (PDF) [file pone.0184193.s001.pdf]
