# Supplementary material for: Combining dispersal, landscape connectivity and habitat suitability to assess climate-induced changes in the distribution of Cunningham’s skink, Egernia cunninghami
Source: PLoS One. 2017 Sep 5;12(9):e0184193. doi: 10.1371/journal.pone.0184193 (PMC5584964; doi:10.1371/journal.pone.0184193)
Supplement: S1 Table — Scores are based-on the tendency of and ease with which Cunningham’s skinks will move through the different cover types. (PDF) [file pone.0184193.s002.pdf]

Supporting Information

S1Table. Mean resistance scores for the land-use and land cover types in the study area as perceived by seven herpetologists (experts). Scores are based-on the tendency of and ease with which Cunningham's skinks will move through the different cover types.

Class ID	Class Description	Resistance score
1	Nature conservation	10
2	other protected areas	10
3	Minimal use	12
4	Grazing native vegetation	15
5	Production forestry	15
6	Grazing modified pastures	25
7	Plantation forestry	15
8	Dryland cropping	25
9	Dryland horticulture	25
10	Land in transition	25
11	Irrigation pasture	30
12	Irrigation cropping	25
13	Irrigation horticulture	25
14	Urban intensive uses including highways	82
15	Intensive anima land/plant production	38
16	Rural residential and farm infrastructure	43
17	mining and waste site	66
18	Large inland bodies	77
