# Supplementary material for: Combining dispersal, landscape connectivity and habitat suitability to assess climate-induced changes in the distribution of Cunningham’s skink, Egernia cunninghami
Source: PLoS One. 2017 Sep 5;12(9):e0184193. doi: 10.1371/journal.pone.0184193 (PMC5584964; doi:10.1371/journal.pone.0184193)
Supplement: S2 Table — (PDF) [file pone.0184193.s003.pdf]

Supporting Information

S2 Table. Least-cost distance (in meters) between occupied suitable grid cells that were projected to become climatically unsuitable and the nearest grid cell projected to retain its suitability or to become suitable for each decadal time interval.

2020-2030	2030-2040	2040-2050	2050-2060	2060-2070
9,576	4,748	17,583	21,883	12,166
13,123	6,085	21,932	25,689	17,353
16,656	6,125	24,005	42,243	24,070
19,700	7,238	24,442	42,747	31,758
24,585	7,893	25,899	51,733	45,057
26,207	9,511	31,785	68,580	59,960
30,353	9,727	36,557	69,560	88,594
44,110	10,002	38,183	88,849	92,257
	11,866	74,564	109,658	
	12,034	86,260	140,983	
	12,102	96,132	169,001	
	25,223	99,100	199,745	
	30,874	143,888		
	33,121	168,963		
	78,441			
	80,957			
